# Erianin Induces Ferroptosis of Renal Cancer Stem Cells *via* Promoting *ALOX12*/*P53* mRNA N6-methyladenosine Modification

**DOI:** 10.7150/jca.81027

**Published:** 2023-01-22

**Authors:** Hongliang Shen, Zixiang Geng, Xiaoli Nie, Te Liu

**Affiliations:** 1 Department of Urology, Beijing Friendship Hospital, Capital Medical University, Beijing 100050, China.; 2 Shuguang Hospital Affiliated to Shanghai University of Traditional Chinese Medicine, Shanghai 200031, China.; 3 Shanghai Geriatric Institute of Chinese Medicine, Shanghai University of Traditional Chinese Medicine, Shanghai 200031, China.

**Keywords:** Renal cancer stem cell, Erianin, N6-methyladenosine (m6A) modification, Ferroptosis, ALOX12/P53

## Abstract

Renal cell carcinoma (RCC) is the most common type of primary renal parenchymal malignancy in adults, with a high degree of malignancy and poor prognosis. Human renal cancer stem cells (HuRCSCs) are reported to be the main cause of drug resistance, metastasis, recurrence, and poor prognosis. Erianin is a low molecular-weight bibenzyl natural product extracted from *Dendrobium chrysotoxum*, which inhibits the *in vitro* and *in vivo* activity of a variety of cancer cells. However, the molecular mechanisms of Erianin's therapeutic effect on HuRCSCs are unknown. Here, we isolated CD44+/CD105+ HuRCSCs from patients with renal cell carcinoma. The experiments confirmed that Erianin significantly inhibited the proliferation, invasion, angiogenesis, and tumorigenesis of HuRCSCs, and induced oxidative stress injury and Fe^2+^ accumulation. qRT-PCR and western blotting showed that Erianin significantly reduced the expression levels of cellular Ferroptosis protective factors, and upregulated the expression of METTL3 and downregulated that of FTO. Dot blotting results indicated that Erianin significantly upregulated the mRNA N6-methyladenosine (m6A) modification of HuRCSCs. The results of RNA immunoprecipitation-PCR also indicated that Erianin significantly enhanced the m6A modification level of the 3' untranslated region of *ALOX12* and *P53* mRNA in HuRCSCs, resulting in increased stability, prolonged half-life, and improved translation activity. In addition, clinical data analysis showed that the expression of *FTO* correlated negatively with adverse events in patient with renal cell carcinoma. Thus, this study suggested that Erianin can induce Ferroptosis in renal cancer stem cells by promoting N6-methyladenosine modification of *ALOX12*/*P53* mRNA, ultimately achieving a therapeutic effect on renal cancer.

## Introduction

Renal cell carcinoma (RCC) is the most common type of primary renal parenchymal malignant tumor in adults. It originates from the proximal convoluted tubule epithelial system. The incidence of RCC in the genitourinary system is second only to bladder cancer, accounting for about 2-3% of adult tumors and 80-90 % of renal malignant tumors 1-3. RCC has high malignancy and poor prognosis, and is a serious threat to human health 1-3. The occurrence of RCC includes a series of pathological and molecular changes in clinical malignant tumors of renal organs 1-3. Clear cell carcinoma and papillary carcinoma (category 1 and 2) account for the majority of RCC 1-3. Recent studies have found that RCC contains stem cell subsets with strong proliferation, invasion, drug resistance, and metastasis abilities, which are termed renal cell carcinoma stem cells (HuRCSCs), which are likely to be the main source of metastasis, recurrence, and poor prognosis in patients with RCC 4, 5. In a previous study, we found that the compound fisetin suppressed Tet methylcytosine dioxygenase 1 (TET1) expression and reduced the 5hmC modification in specific loci in the promoters of *CCNY* (encoding cyclin Y)/CDK16 (encoding cyclin dependent kinase 16) in HuRSCs, which inhibited the transcription of these genes, causing cell cycle arrest and ultimately inhibiting renal cancer stem cell activity 4. Meanwhile, we reported that suppressed expression of the long non-coding RNA *HOTAIR* inhibited proliferation and tumorigenicity of renal carcinoma cells 5. Thus, epigenetic regulation significantly affects the malignant degree of renal cancer cells.

Erianin is a low-molecular-weight bibenzyl natural product extracted from *Dendrobium chrysotoxum* 6-8. Initial reports found that Erianin can be used as an antipyretic and analgesic agent to inhibit indoleamine 2,3-dioxygenase-induced tumor angiogenesis 7. Later studies found that Erianin can inhibit tumor cell cycle progression and induce tumor death by inhibiting BCL2 apoptosis regulator (Bcl-2) and extracellular regulated kinase (ERK)1/2 and promoting BCL2 associated X, apoptosis regulator (Bax) and caspase-3 expression 9-11. Erianin can inhibit the proliferation and induce apoptosis of colon cancer, bladder cancer, liver cancer, gastric cancer and melanoma 9, 12.

Ferroptosis is a novel iron‑dependent programmed cell death 13-16. The action of ferrous iron or esterase catalyzes the high expression of unsaturated fatty acids causing lipid peroxidation could induce ferroptosis 15, 17-20. Currently, many studies have reported that some bioactive compouns or small molecule drugs can significantly inhibit cancer cells' self-proliferation, division, and invasion activities via promoting ferroptosis 13, 14, 16, 17, 19. Chen et al. found that the natural product, Erianin, exerted its anticancer effects by inducing Ca2+/calmodulin (CaM)-dependent Ferroptosis and inhibiting cell migration, and Erianin might serve as a prospective compound to treat lung cancer 21. However, it has not been reported whether Erianin can induce the occurrence of Ferroptosis in HuRCSCs and its underlying molecular biological mechanism.

RNA N-6 methyladenosine (m6A) is a methylation modification of N atoms at position 6 of RNA adenine 22-27. RNA m6A methylation modification exists widely in most eukaryotic species (from yeast, plant, and fruit fly to mammals) and in viral mRNA, playing a key role in posttranscriptional mRNA regulation and metabolism 22-27. Methyltransferase 14, N6-adenosine-methyltransferase subunit (METTL14) and methyltransferase 3, N6-adenosine-methyltransferase complex catalytic subunit (METTL3) are two components of m6A methyltransferase complexes. These two proteins can form stable complexes at a ratio of 1:1 to complete RNA m6A modification, belonging to the “Writers” 22-27. The fat mass and obesity-associated protein (FTO) removes methylation of RNA m6A, acting as an “Eraser” 22-27. Therefore, RNA m6A modification is a dynamic and reversible enzymatic reaction 22-27. Studies have suggested that the RNA m6A modification can improve the stability of mRNA, increase its transcription and translation activities, promote tumor occurrence and invasion, and improve the reprogramming efficiency of stem cells 22-27. However, the regulatory mechanism of dynamic m6A modification of RNA during ferroptosis has not been determined.

Based on the above evidence, we aimed to isolate CD44+/CD105+ HuRCSCs from tissue samples of patients with RCC. *In vitro* and* in vivo*, we confirmed that Erianin, through gene mRNA m6A modification of key gene enzyme expression, regulated ferroptosis-related gene mRNA m6A modification and its mRNA stability, eventually inducing HuRCSCs ferroptosis via epigenetic mechanism.

## Materials and Methods

A detailed description of all materials and methods can be found in supplementary data.

### CD44+/CD105+ HuRCSCs isolation and culture

CD44+/CD105+ HuRCSCs were isolated according to a previously published methods 4. Briefly, human RCC tissues from four patients were digested using trypsin (containing 0.02% EDTA-Na) at 37 °C for 30 minutes and the reaction was terminated using cell culture medium containing 15% fetal bovine serum (FBS). The volume of the cell suspension was adjusted and 4 μl fluorescein isothiocyanate (FITC)-labelled rabbit anti‑human CD44 monoclonal antibody and Cy3-labelled rabbit anti-human CD105+ antibody (eBioscience, San Diego, CA, USA) were added to 100 μl of cell suspension and incubated in the dark at 4 °C for 30 minutes. Pre-cooled phosphate buffered saline (PBS) was used to readjust the volume of the cell suspension to 500 μl. A flow cytometer (BD FACSAria, BD Biosciences San Jose, CA, USA) was used to select CD44+/CD105+ HuRCSCs. All cells were resuspended in complete cancer stem cell culture medium: Dulbecco's modified Eagle's medium (DMEM:F12 (HyClone, Logan, UT, USA), supplemented with 10 ng/mL basic fibroblast growth factor, 10 ng/mL epidermal growth factor, 5 μg/mL insulin, 1% bovine serum albumin (BSA) and 5% knockout serum replacement (KnockOut SR) (all from Gibco, Grand Island , NY, USA). The study protocol was approved by the Regional Ethics Committee of Shanghai Geriatric Institute of Chinese Medicine, Shanghai University of Traditional Chinese Medicine (Permission No.: SHAGE-E-202114), in accordance with the 2008 Helsinki declaration.

### 3-(4,5-dimethylthiazol-2-yl)-2,5-diphenyltetrazolium-bromide (MTT) assay

Briefly, 2000 cells/ml of each group were seeded in a 96-well plate. After 24 h, 10 μl of MTT solution (Sigma-Aldrich, St. Louis, MO, USA) was added to each group of cells and incubated at 37 °C for 3 h. The medium was discarded, 150 μl of dimethyl sulfoxide (DMSO) (Sigma-Aldrich) was added to each well, and the plate was shaken for 15 s to mix well. The culture plate was placed in a microplate reader to record the absorbance value at 450 nm. The formula for calculating the cell proliferation inhibition rate (%) is (1-OD value of experimental group of cells - blank/OD value of control group of cells - blank) × 100%.

### RNA extraction and Quantitative real-time reverse transcription PCR (qRT-PCR)

According to the instructions of the RNAprep pure Tissue Kit (TIANGEN Biotech (Beijing) Co., Ltd., Beijing, China), about 20 mg of human tissue samples were taken, added with 800 μl lysis buffer, ground, and homogenized. The supernatant was retained, added with 200 μl of chloroform, mixed by inversion, and centrifuged at 4 °C, 13 400 ×* g* for 15 min. Two volumes of anhydrous ethanol times were added to the supernatant, mixed by inversion, and centrifuged at 4 °C, 13400 ×* g*, for 30 min. RNA pellet was resuspended with 500 μl 75 % ethanol centrifuged at 4 °C, 13400 × *g* for 5 min. All the liquid was removed and the RNA pellet was fully dissolved in 300 μl of diethyl pyrocarbonate (DEPC) water. The ratio of OD260/OD280 (generally controlled between 1.8 and 2.0) was detected for 1 μl of the RNA solution to determine the purity and total concentration of RNA. Total RNA was treated with DNase I (Sigma-Aldrich), quantified, and reverse transcribed into cDNA using the ReverTra Ace-α First Strand cDNA Synthesis Kit (TOYOBO). The qRT-PCR was performed with a RealPlex4 real-time PCR detection system from Eppendorf Co. Ltd. (Germany). SYBR Green Real-Time PCR Master Mix (TOYOBO) was used as the fluorescent dye in the nucleic acid amplification. qRT-PCR was completed with 40 amplification cycles as follows: denaturation at 95 °C for 15s, annealing at 58 °C for 30s, and extension at 72 °C for 42s. The relative gene expression levelswere calculated using the 2^-ΔΔCt^ method (ΔCt = Ct_genes - Ct_18sRNA; ΔΔCt = ΔCt_all_groups - ΔCt_blankcontrol_group). The mRNA expression levels were normalised to the expression level of 18s rRNA.

### Western blotting

In brief, the total proteins of each group were subjected to 12% denaturing sodium dodecylsulfate polyacrylamide gel electrophoresis (SDS-PAGE), and transferred to a polyvinylidene fluoride (PVDF) membrane (Millipore, Bedford, MA, USA) after completion. After blocking and washing, primary antibodies were added and incubated at 37 °C for 45 min. After sufficient washing, the secondary antibodies were added incubated at 37 °C for 45 min. The membrane was washed four times with Tris-buffered saline-Tween20 (TBST) at room temperature for 14 min each time. Then, Sigma-Aldrich Chemical was added and the immunoreactive protein bands were developed using an Enhanced Chemiluminescence (ECL) kit (Pierce Biotechnology, Rockford, IL, USA).

### *In vivo* xenograft experiments

BALB/C^nu/nu^ mice aged 6-7 weeks and weighing about 20 g were used in the experiment. The BALB/C^nu/nu^ mice were administered with approximately 1 × 10^5^ cells in the log phase. Each experimental group consisted of four mice. After 2 months, the mice were sacrificed, and their tumors were excised. The tumour weight was measured and the tumor volume was calculated according to the formula: Tumor volume (mm^3^) = (wh^2^)/2, where w is the longest axis (mm) and h is the shortest axis (mm). The animal study was performed at the Shanghai University of Traditional Chinese Medicine with approval from the Institutional Animal Care and Use Committee in accordance with the institutional guidelines. And, all animal experiments complyed with the ARRIVE guidelines and were carried out in accordance with the National Institutes of Health guide for the care and use of Laboratory animals (NIH Publications No. 8023, revised 1978).

### Bioinformatic prediction and analysis

A total of 522 patients with renal cell carcinoma patients(T) and 99 non-renal cell carcinoma patients (N) from the Gene Expression Profiling Interactive Analysis (http://gepia.cancer-pku.cn/index.html), GEPIA, were included in the study patient cohorts. The data for the above patient cohorts were used in gene expression profile analysis, pathological stage plot analysis, multiple gene comparison analysis, and gene correlation analysis using the GEPIA online tool.

### Statistical analysis

Each experiment was performed as least three times; data are presented as the mean ± the standard error (SE) where applicable. Differences were evaluated using Student's t-tests. P values < 0.05 were considered statistically significant. With respect tothe ANOVA and limma options, genes with a |log2FC| cutoff > 1 and q < 0.01 relative to pre-set thresholds were considered to be differently expressed genes (DEGs).

## Results

### Erianin significantly reduced the *in vitro* activity of HuRCSCs

**Figure 1:**
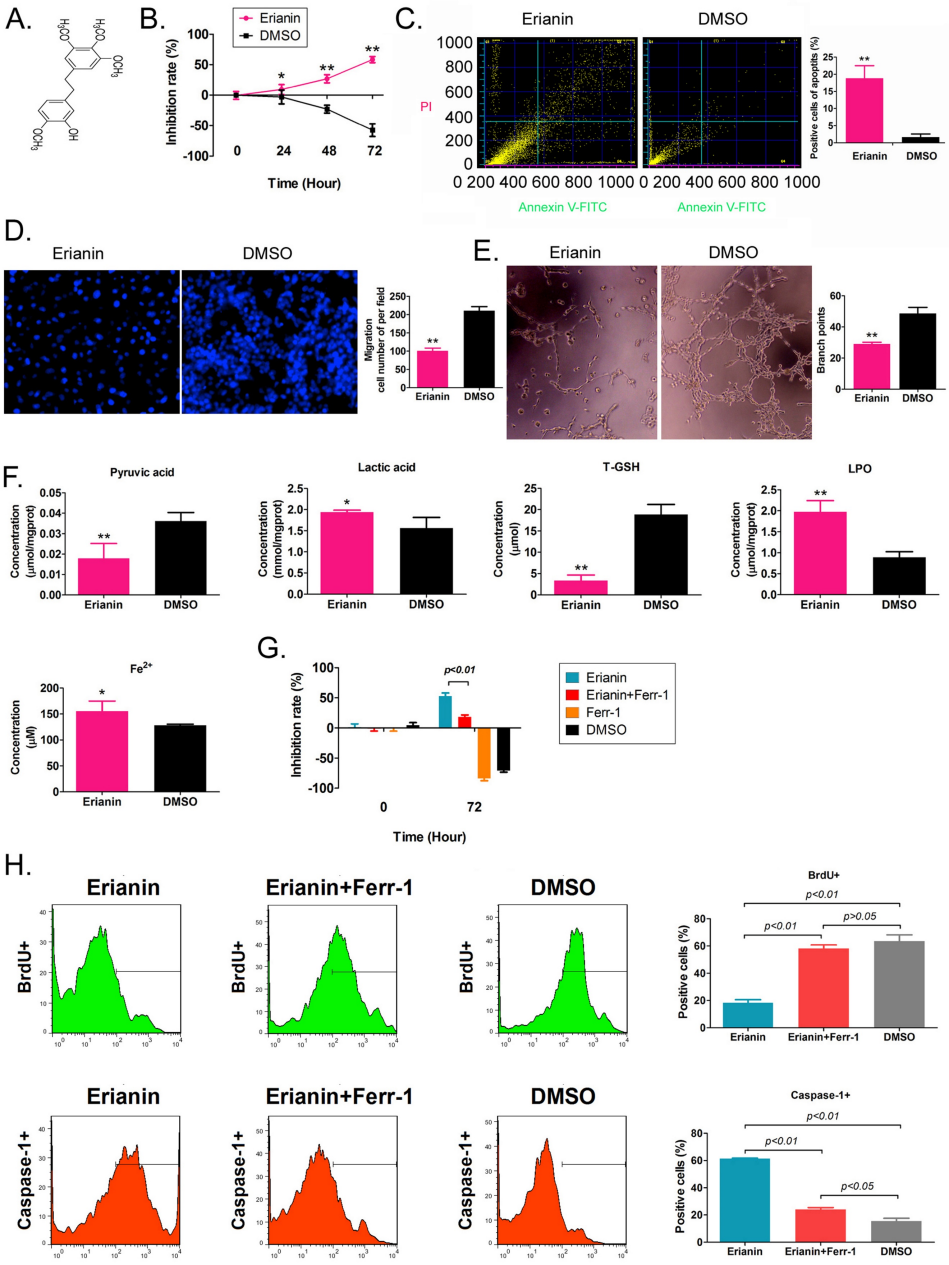
** Erianin significantly decreased the activity of HuRCSCs *in vitro*. (A)** The molecular structure of Erianin. **(B)** MTT assay results showing that Erianin significantly inhibited the proliferation of HuRCSCs *in vitro*. **p < 0.01 *vs*. DMSO; *p < 0.05 *vs*. DMSO; t test; n = 4. **(C)** Flow cytometry analysis showing that Erianin induced HuRCSC apoptosis *in vitro*. **p < 0.01 *vs*. DMSO; t test; n = 4. **(D)** Transwell chamber results showing that Erianin significantly inhibited the migration of HuRCSCs in the external matrix. **p < 0.01 *vs*. DMSO; t test; n = 4. **(E)** Erianin significantly inhibited the angiogenesis of HUVECs in the external matrix. **p < 0.01 *vs*. DMSO; t test; n = 4. **(F)** Biochemical assay showing that Erianin significantly downregulated pyruvate and T-GSH concentrations and upregulated lactic acid, LPO, and Fe2+ concentrations in HuRCSCs. **p < 0.01 *vs*. DMSO; *p < 0.05 *vs*. DMSO; t test; n = 4. **(G)** The MTT results showing that the cell inhibition rate of Erianin + Ferr-1 treatment group was significantly decreased. **(H)** Flow cytometry analysis results indicated that the precentage of BrdU+ HuRCSCs of Erianin treated group was significantly decreased. (I) Flow cytometry analysis results indicated that the precentage of BrdU+ HuRCSCs of Erianin treated group was significantly elevated.

Erianin is an active substance from Dendrobium *chrysotoxum*, comprising a low molecular weight bibenzyl natural product (Figure 1A). According to the previous study 21, the Erianin concentration of 50 nM was used to treated to HuRCSCs. The results of the MTT assay showed that Erianin treatment inhibited the proliferation of HuRCSCs significantly increased in a treatment time-dependent manner (Figure 1B). The results of flow cytometry showed that Erianin significantly increased the apoptosis rate of HuRCSCs (Figure 1C). The results of the Transwell chamber experiment showed that Erianin significantly inhibited the migration of HuRCSCs into the external matrix (Figure 1D). Moreover, the results of the matrix gel angiogenesis experiment also showed that Erianin could significantly weaken the ability of HUVECs to form blood vessels in the matrix gel (Figure 1E). In addition, the biochemical test results showed that after Erianin treatment of HuRCSCs, the concentrations of intracellular lactic acid, lipid peroxide (LPO) and Fe2+ were increased, and the concentrations of pyruvic acid and total glutathione (T-GSH) were decreased (Figure 1F). Meanwhile, the MTT assay was used to determine the weakening effect on ferroptosis of Ferrostatin-1 (Ferr-1, antagonist of ferroptosis) combined with Erianin treatment for HuRCSCs. The MTT results showed that the cell inhibition rate of Erianin + Ferr-1 treatment group was significantly lower than it in only Erianin treatment group (Figure 1G). The BrdU incorporation assay and cell immunofluorescence staining combined with flow cytometry is used to determine the cell proliferation and necroptosis of Erianin treatment of HuRCSCs. The results indicated that the precentage of BrdU+ HuRCSCs (the biomarker of cell proliferation) of Erianin treated group was significantly lower than it in Erianin + Ferr-1 treated group or DMSO group (Figure 1H). The results revealed that the precentage of Caspase-1+ HuRCSCs (the biomarker of necroptosis) of Erianin treated group was significantly higher than it in Erianin + Ferr-1 treated group or DMSO group (Figure 1I). These results showed that Erianin significantly reduced the *in vitro* activity of HuRCSCs by inducing oxidative stress injury and weakening their energy metabolism activity.

### Erianin promotes the high expression of genes related to ferroptosis in HuRCSCs

**Figure 2:**
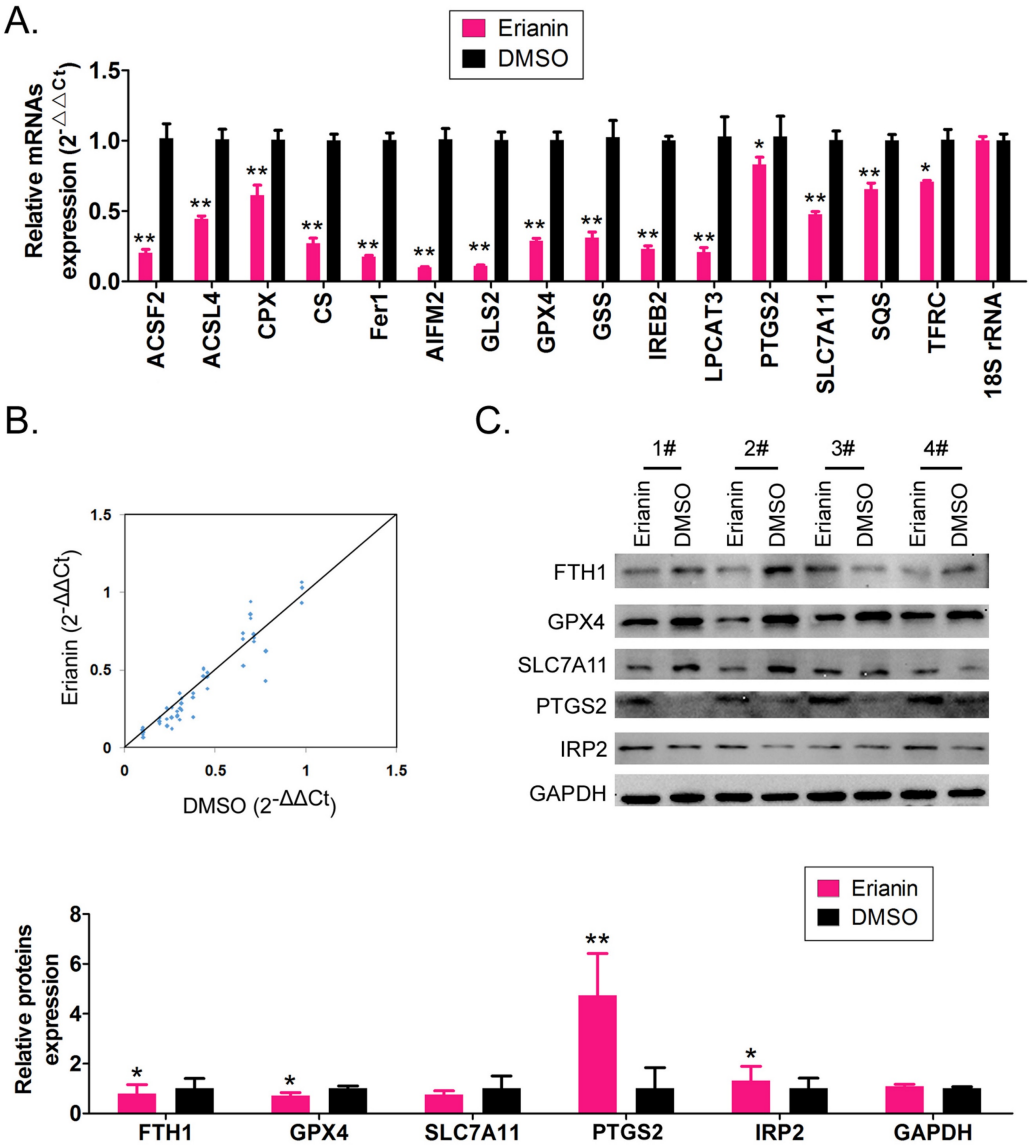
** Erianin regulates the expression level of iron death-related factors. (A)** qRT-PCR results indicating that Erianin downregulates the mRNA expression of ferroptosis protection genes in HuRCSCs. **p < 0.01 *vs*. DMSO; *p < 0.05 *vs*. DMSO; t test; n = 4. **(B)** qRT-PCR results indicating that the mRNA expression levels of multiple iron death-related genes were significantly different between the two groups. **(C)** qRT-PCR results showing that the mRNA expression levels of multiple ferroptosis-related genes were significantly different between the two groups. **p < 0.01 *vs*. DMSO; *p < 0.05 *vs*. DMSO; t test; n = 4.

The qRT-PCR results showed that after Erianin treatment of HuRCSCs, the mRNA expression levels of intracellular ferroptosis inhibitory genes *GPX4*, *AIFM2* (also known as *FSP1* (encoding atlastin apoptosis inducing factor mitochondria associated 2)), *IREB2* (encoding iron responsive element binding protein 2), *GSS* (encoding glutathione synthetase), *SLC7A11* (encoding solute carrier family 7 member 11), *SQS* (encoding squalene synthase), and CS (encoding citrate synthase) were significantly lower than those in the control group (DMSO treatment group) (Figure 2A, 2B). The western blotting results also showed that after Erianin treatment of HuRCSCs, the levels of intracellular ferroptosis inhibitory proteins GPX4, ferritin heavy chain 1 (FTH1), and SLC7A11 were significantly lower than those in the DMSO control group, while the levels of ferroptosis promoting proteins, prostaglandin-endoperoxide synthase 2 (PTGS2) and iron responsive element binding protein 2 (IRP2), were significantly increased (Figure 2C).The experimental data suggested that Erianin could significantly increase the expression of positive regulatory factors for ferroptosis of HuRCSCs, but inhibited the expression of negative regulatory factors.

### Erianin promotes m6A methylation modification of the mRNA encoding the key regulatory factors ALOX12 and p53 in the whole RNA and in the ferroptosis signal transduction pathway of HuRCSCs

**Figure 3:**
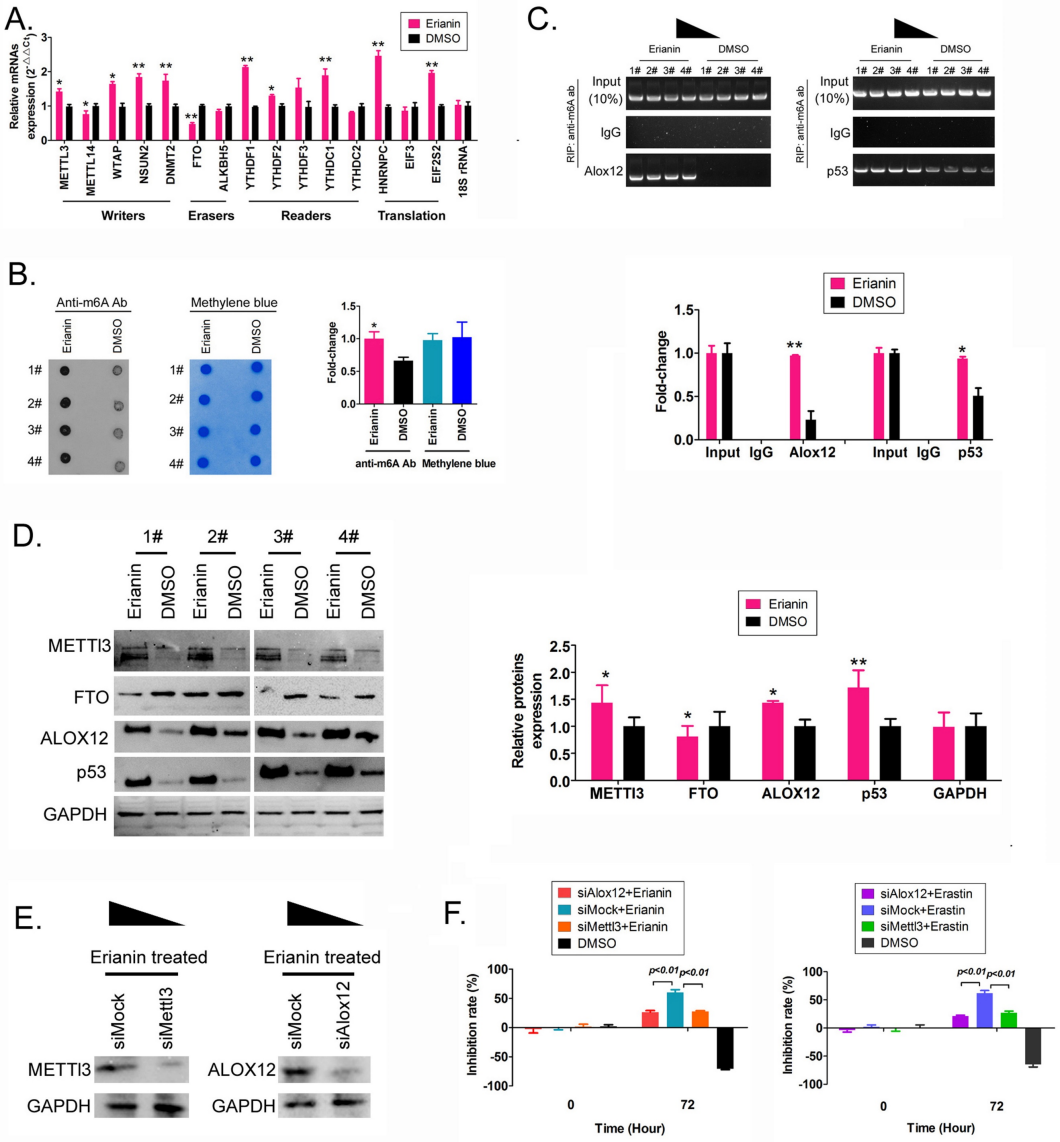
** Erianin promotes the overall RNA m6A modification of HuRCSCs. (A)** qRT‑PCR results indicating that Erianin upregulated *METTL3* and downregulated *FTO* mRNA expression. **p < 0.01 *vs.* DMSO; *p < 0.05 *vs.* DMSO; t test; n = 4. **(B)** Dot blotting results showing that Erianin downregulated the m6A modification of HuRCSCs mRNA. *p < 0.05 *vs*. DMSO; t test; n = 4. **(C)** The results of RIP-PCR indicating that Erianin promoted the m6A modification level of the ALOX12 and P53 mRNAs. **p < 0.01 *vs*. DMSO; *p < 0.05* vs*. DMSO; t test; n = 4. **(D)** Western blotting results showed that Erianin significantly upregulated METTL3, ALOX12, and p53 levels and downregulated the level of FTO protein. **p < 0.01 *vs*. DMSO; *p < 0.05 *vs*. DMSO; t test; n = 4. (D) Western blotting results on siAlox12 and siMettl3 transfected group. **(E)** The results of MTT assay.

First, we detected the differences in the expression levels of RNA m6A methylation writing enzymes, erasing enzymes, reading enzymes, and translation enzymes in each group. The qRT-PCR results showed that after Erianin treatment of HuRCSCs, the expression levels of *METTL3*, *WTAP* (encoding WT1 associated protein), *NSUN2* (encoding NOP2/Sun RNA methyltransferase 2), *DNMT2* (encoding DNA methyltransferase-2) and other RNA methylation “Writers” were significantly higher than those in the control group, while the expression level of the RNA methylation “Eraser” *FTO* was significantly decreased (Figure 3A). Dot blotting results showed that the overall mRNA m6A modification level of HuRCSCs treated with Erianin was significantly higher than that of the control group (Figure 3B). In addition, the results of RIP-PCR showed that after HuRCSCs were treated with Erianin, the specific products of 3' untranslated region (UTR) of *ALOX12* (encoding arachidonate 12‑Lipoxygenase, 12S Type) and *P53* (encoding tumor protein p53) mRNA could be amplified by PCR in the complex associated with anti-m6A antibody (Figure 3C). In the control group, in the complex associated with anti-m6A antibody, it was almost impossible to PCR amplify the specific products of the 3' UTRs of the above factors (Figure 3C).

Finally, the western blotting results showed that the levels of METTL3, ALOX12, and p53 proteins in HuRCSCs treated with Erianin were significantly higher than those in the control group, while the level of FTO showed the opposite trend (Figure 3D). In addition, in order to confirm the correlation between ALOX12 and METTL3 induced RNA m6A modification and cell ferroptosis, the siRNAs were used to knockdown the expression of endogenous ALOX12 and METTL3 in HuRCSCs (Figure 3E). The results of MTT assay showed that the cell inhibition rate of siAlox12+Erianin treatment group was significantly lower than it in control group (siMock+Erianin), and the cell inhibition rate of siMettl3+Erianin treatment group was also significantly lower than it in control group (Figure 3F). Besides, the MTT assay was used to determine the influencing effect on ferroptosis of Erastin (agonist of ferroptosis) combined with siRNAs treatment for HuRCSCs. The results showed that the cell inhibition rate of siAlox12+Erastin or siMettl3+Erastin treatment group was significantly lower than it in siMock+Erastin treatment group (Figure 3F). Collectively, these results showed that on the one hand, Erianin could increase the overall RNA m6A methylation level of HuRCSCs by promoting the expression of RNA m6A methylases, and on the other hand, Erianin could increase its stability and expression level by promoting m6A methylation at specific sites in the 3' UTR of *ALOX12* and *P53* mRNA, the key regulators of the ferroptosis pathway.

### Erianin inhibits the tumorigenicity of HuRCSCs *in vivo* by promoting ferroptosis-related protein expression

**Figure 4:**
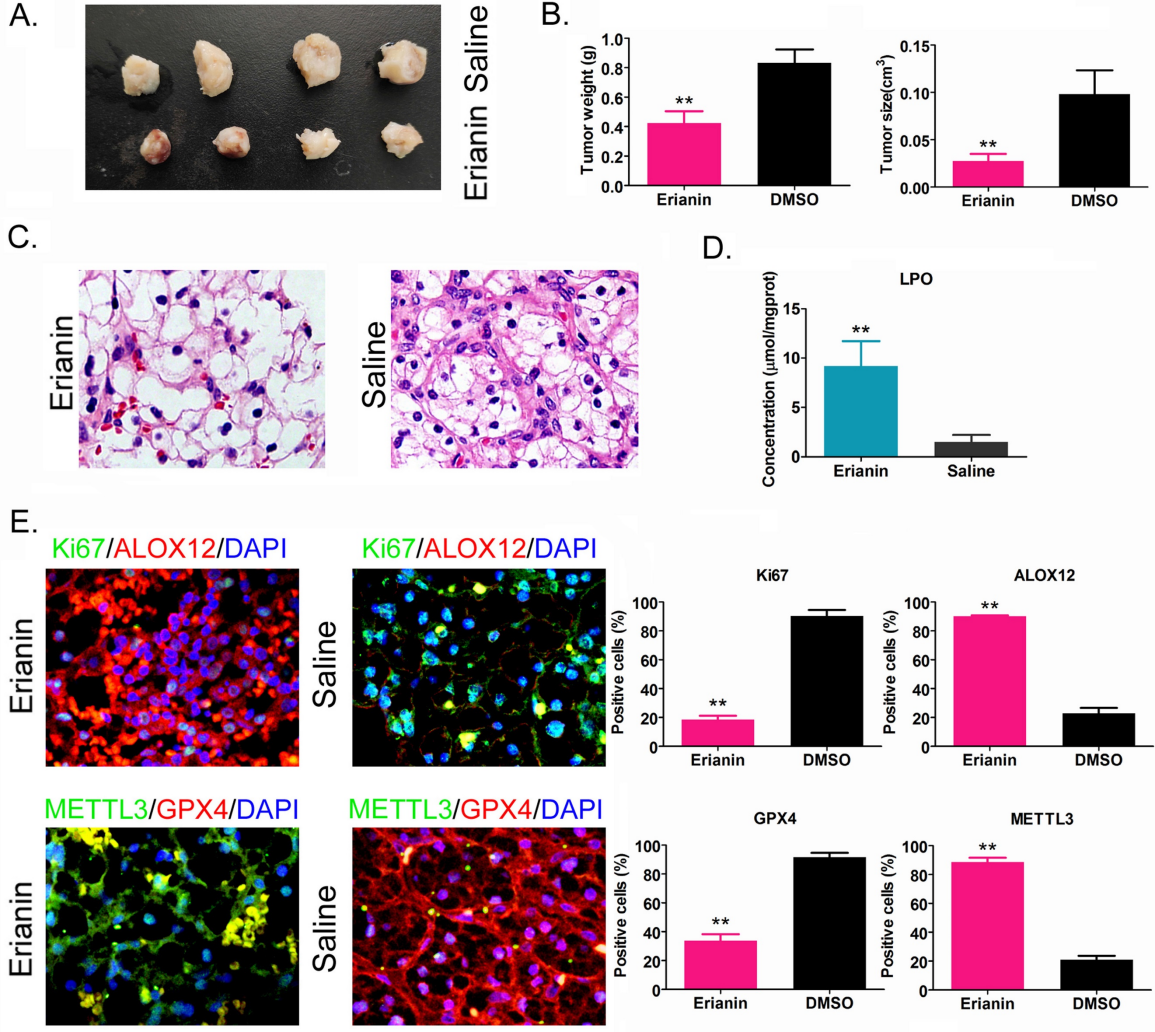
** Erianin Inhibits Tumorigenicity of HuRCSCs in Nude Mice. (A)** Morphology of the dorsal tumors of tumor-bearing mice in each group. **(B)** Effect of Erianin on tumor volume and weight in nude mice. *p < 0.05 *vs*. Saline; t test; n = 4. **(C)** H & E staining confirming renal clear cell carcinoma in each group. **(D)** Biochemical assay on LPO. *p < 0.05 *vs*. Saline; t test; n = 4. **(E)** The results of immunohistochemical staining showed that Erianin intervention could significantly upregulate the levels of METTL3, ALOX12 and others in tumor tissues, and downregulated the levels of Ki67 and GPX4.

HuRCSCs were inoculated onto the back of nude mice, and Erianin was injected intraperitoneally every 2 days. The nude mice were sacrificed around the ninth week. Naked eye observation showed that the tumors on the back of the nude mice in the Erianin injection group were significantly smaller than those in the control group (Figure 4A). Tumor tissue was isolated from the back of nude mice in each group. The weight and volume of the tumor tissue from the Erianin intervention group were significantly lower than those in the control group (Figure 4B). H & E staining showed that although the two groups of tumors were consistent with the pathological characteristics of clear cell renal cell carcinoma, the tumors from the Erianin intervention group had obvious vascular rupture and cell swelling (Figure 4C). The results of biochemical test showed that the concentration of tissue lipid peroxide (LPO) on Erianin intervention group was elevated significantly compared to it in the control group (Figure 4D). Immunohistochemical staining showed that the expression levels of marker of proliferation Ki-67 (Ki67) and GPX4 in the Erianin intervention group were significantly lower than those in the control group, while the expression levels of ALOX12 and METTL3 were significantly higher than those in the control group (Figure 4E). The experimental results suggested that Erianin promoted the expression of ferroptosis-related proteins and weakened the tumorigenicity of HuRCSCs in nude mice by regulating the expression of m6A methylation modification enzymes.

### Correlation between *FTO* gene expression and clinical prognosis of renal cell carcinoma

**Figure 5:**
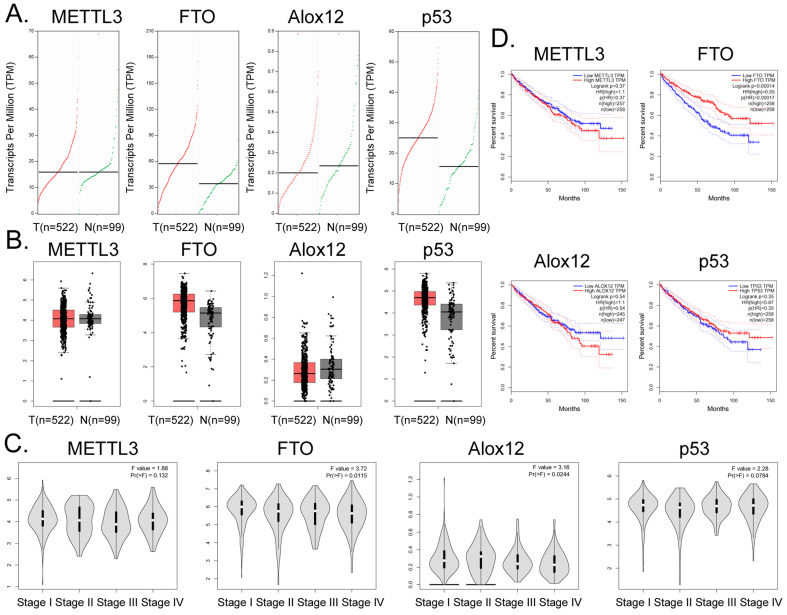
** Correlation between *FTO* gene expression and clinical prognosis of renal cell carcinoma. (A)** Numbers of transcripts of *FTO* and *P53* in tumor tissues were significantly higher than those in normal tissues. **(B)** The mRNA expression levels of *FTO* and *P53* were significantly higher in tumor tissues than in normal controls. **(C)** There was no statistically significant difference in the expression levels of *METTL3*, *FTO*, ALOX12, *P53*, and other genes in all stages of renal cell carcinoma. **(D)** The statistical results of the survival curve for patients with tumors indicated that the survival period of patients with renal clear cell carcinoma with high *FTO* expression was significantly higher than that of patients with low *FTO* expression.

Bioinformatic analysis of 522 tumor tissues from patients with clear cell renal cell carcinoma and 99 tissue samples from non-tumor diseases in the online database GEPIA (http://gepia.cancer-pku.cn/) showed that the transcription copy numbers of *FTO* and *P53* in tumor tissues were significantly higher than those in normal tissues (Figure 5A), while the transcription copy number of *ALOX12* in tumor tissues was slightly lower than that in normal tissues (Figure 5A). The mRNA expression levels of *FTO* and *P53* in tumor tissue samples were significantly higher than those in normal control group samples, while the mRNA expression level of *ALOX12* showed the opposite results (Figure 5B). However, there was no statistically significant difference in the expression levels of* METTL3*, *FTO*, *ALOX12*, *P53* and other genes among all the stages of renal cell carcinoma (Figure 5C). In addition, the statistical results of the survival curve of patients with tumors indicated that the survival period of patients with RCC with high *FTO* expression was significantly longer than that of patients with low *FTO* expression (Figure 5D). Therefore, clinical data analysis showed that the expression level of *FTO* correlated positively with the survival patients with RCC.

## Discussion

To date, many studies have pointed out that tumor tissue contains a special group of cell subsets, namely cancer stem cells 1, 4, 5, 28-30. Cancer stem cells have strong proliferation and invasion abilities, increased tolerance to chemotherapeutic drugs, and easily induce tumor metastasis and recurrence, which directly promotes poor prognosis of patients with tumors 1, 4, 5, 28. *In vivo* and *in vitro* experiments have shown that the drug resistance of cancer stem cells is much stronger than that of ordinary tumor cells, and they are resistant to a wide range of chemotherapeutic drugs, involving, for example, platinum drugs, paclitaxel, and gemcitabine 29, 30. Inhibition of the drug resistance of cancer stem cells is the key to blocking tumor cell reactivation and tumor recurrence. The limitations of traditional chemotherapeutic drugs in killing cancer stem cells have led many researchers to investigate natural products, hoping to find substances that can inhibit cancer stem cells. In the present study, we chose to investigate Erianin. Cell‑based experiments showed that Erianin significantly inhibited the proliferation and invasion of RCC stem cells *in vitro* and *in vivo*. These encouraging results suggested that Erianin has potential as an effective small molecule drug to inhibit cancer stem cells. However, it is necessary to clarify the molecular biological mechanism by which Erianin inhibits cancer stem cells. In this study, we approached this from two directions.

On the one hand, we attempted to clarify the mechanism by which Erianin promoted ferroptosis of RCC stem cells. The occurrence of ferroptosis is closely related to the levels of GPX4, AIFM2, Fe2+, glutamine and lipid peroxide (LPO). Our results suggested that Erianin inhibits GPX4 expression and promotes LPO and Fe2+ accumulation in RCC stem cells, i.e., Erianin might promote drug toxicity by inducing ferroptosis in RCC stem cells. We also referred to recent studies on ferroptosis. For example, Chu et al. found that p53 activates ALOX12 indirectly via transcriptional repression of *SLC7A11*, resulting in ALOX12-dependent ferroptosis upon reactive oxygen species (ROS) stress 31. This prompted us to investigate the effects of Erianin on ALOX12 and p53 expression. We confirmed that the expression levels of ALOX12 and p53 in Erianin-treated cells increased significantly. We speculated that the mechanism by Erianin induces ferroptosis of RCC stem cells is consistent with the results of the study of Chu et al. Furthermore, Chu et al. reported that ALOX12 was dispensable for ferroptosis induced by erastin or GPX4 inhibitors 31. Thus, their study identified an ALOX12-mediated ferroptosis pathway that was critical for p53-dependent tumor suppression 31. According to the results reported by Chu et al., an increase in ALOX12 and a decrease in GPX4 can induce ferroptosis, and both are relatively independent pathways. However, our study found that Erianin could inhibit the expression of GPX4 and promote the expression of ALOX12 in RCC stem cells. This result suggested that Erianin-induced ferroptosis in cancer stem cells is likely to involve multiple pathways and targets.

**Figure 6:**
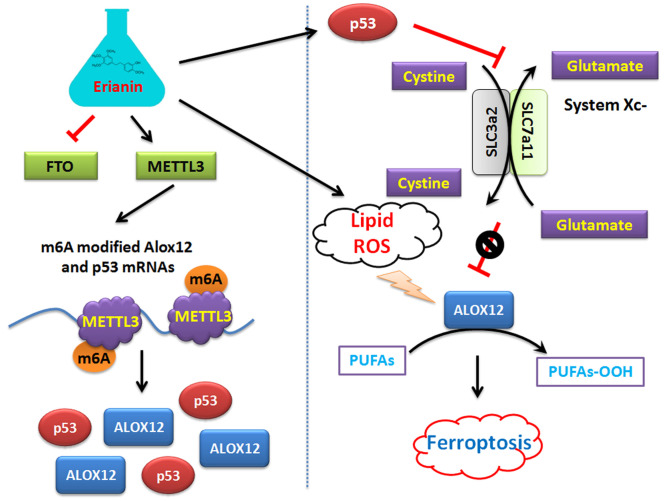
Mechanism by which Erianin promotes the m6A modification of *ALOX12* / *P53* mRNA to induce ferroptosis of renal cell carcinoma stem cells.

On the other hand, this study explained the epigenetic mechanism by which Erianin maintained the stable expression of members of the ALOX12/p53 signaling axis and promoted ferroptosis in HuRCSCs at the level of RNA methylation modification. RNA N-6 methyladenosine (m6A) is a methylation modification that occurs on the sixth nitrogen atom (N) of RNA adenine. M6A methylation modification of RNA exists widely in most eukaryotic species (from yeast and plants to fruit flies and mammals) and in viral mRNA, and plays a key role in posttranscriptional mRNA regulation and metabolism 32-36. The m6A methyltransferases METTL14 and METTL3 are two components of the m6A methyltransferase complexes. These two proteins can form stable complexes at a ratio of 1:1 to complete RNA m6A modification, belonging to the “Writers” group of enzymes 35, 37-39. FTO removes m6A methylation of RNA, acting as an “Eraser” 16, 19, 35, 37-40. Therefore, RNA m6A modification is a dynamic, reversible enzymatic reaction 35, 37-39. Studies have suggested that RNA m6A modification can improve the stability of mRNA, increase its transcription and translation activities, promote tumor occurrence and invasion, and improve the reprogramming efficiency of stem cells 32-39. Meyer et al. and Dominissini et al. used the same m6A-specific binding immunoprecipitation high-throughput sequencing method to determine human and mouse genes, respectively, and studied the distribution of RNA m6A in the whole transcriptome. The results showed that the RNA m6A modification was mainly distributed near the termination codons of the 3'-UTR and coding region (CDS) of mRNA, which proved that the distribution of the m6A modification in human and mouse was highly conserved, and the RNA m6A modification could improve the stability of mRNA 26, 32, 33, 37. Further studies showed that m6A was mainly enriched in the vicinity of mRNA termination codon, 3' UTRs, and exons of mRNA internal minister, and the main conserved sequences were G (m6A) C (70%) or A (m6A) C (30%) 32, 34, 40. There are some reports regarding the mechanism of ferroptosis induced by m6A. Ma et al. reported that the m6A reader YT521-B homology containing 2 (YTHDC2) is a powerful endogenous ferroptosis inducer and targeting the solute carrier 3A2 (SLC3A2) subunit of system XC- is essential for this process 17. Meanwhile, Shen et al. found that the m6A modification appears to trigger autophagy activation by stabilizing *BECN1* mRNA (encoding beclin 1), which might be the potential mechanism for m6A modification-enhanced ferroptosis of hepatic stellate cells 16. In addition, YTH N6‑methyladenosine RNA binding protein 1 (YTHDF1) was identified as a key m6A reader protein for *BECN1* mRNA stability, and knockdown of *YTHDF1* prevented *BECN1* plasmid-induced ferroptosis of hepatic stellate cells 16. In addition, Sun et al. indicated that nuclear factor kappa B (NF-κB) activating protein (NKAP), an RNA‑binding protein, could protect glioblastoma cells from ferroptosis by promoting *SLC7A11* mRNA splicing in an m6A-dependent manner 20. However, whether Erianin can induce changes in the overall RNA m6A modification of tumor cells has not been reported. According to the above clues, we detected the expression of m6A modification-related enzymes before and after Erianin treatment of HuRCSCs. The results suggested that the methylation transferase METTL3 was highly expressed and the methylation erase FTO was poorly expressed. This result suggested that Erianin was likely to promote the overall methylation modification of tumor cell RNA. Subsequently, we detected the m6A modification in the specific region of the 3' UTR of the mRNAs encoding key factors in the of ALOX12/p53 signaling axis, which is closely related to ferroptosis. We found that m6A modification in the 3' UTR specific regions of *ALOX12* and *P53* mRNA was very low in the control group, whereas the m6A modification level of the above genes in tumor cells increased significantly after Erianin treatment (Figure 6). Therefore, we believe that Erianin induces an increase in the m6A methylation modification of multiple genes by promoting the expression of m6A methylation transferase METTL3 in tumor cells.

## Supplementary Material

Supplementary materials and methods.

## Abbreviations

m6A: N6-methyladenosine
HuRCSCs: Human renal cancer stem cells
TET1: Tet methylcytosine dioxygenase 1
ERK: Extracellular regulated kinase
MTT: 2.2 3-(4,5-dimethylthiazol-2-yl)-2,5-diphenyltetrazolium-bromide
DMSO: Dimethyl sulfoxide
qRT-PCR: Quantitative real-time reverse transcription PCR
DEPC: Diethyl pyrocarbonate
SDS-PAGE: Sodium dodecylsulfate polyacrylamide gel electrophoresis
PVDF: Polyvinylidene fluoride
3' UTR: 3' untranslated region
ROS: Reactive oxygen species
RIP-PCR: RNA immunoprecipitation PCR
H&E staining: Hematoxylin and eosin staining
LPO: Lipid peroxidation
ATP: Adenosine triphosphate
