# Supplementary material for: Erianin Induces Ferroptosis of Renal Cancer Stem Cells via Promoting ALOX12/P53 mRNA N6-methyladenosine Modification
Source: J Cancer. 2023 Jan 22;14(3):367–78. doi: 10.7150/jca.81027 (PMC9969579; doi:10.7150/jca.81027)
Supplement: Supplementary file 1 — Supplementary materials and methods. [file jcav14p0367s1.pdf]

2. Materials and Methods

2.1 CD44+/CD105+ HuRCSCs isolation and culture

CD44+/CD105+ HuRCSCs were isolated according to a previously published methods [1]. Briefly, human RCC tissues from four patients were digested using trypsin (containing 0.02% EDTA-Na) at 37 ℃ for 30 minutes and the reaction was terminated using cell culture medium containing 15% fetal bovine serum (FBS). The volume of the cell suspension was adjusted and 4 μl fluorescein isothiocyanate (FITC)-labelled rabbit anti-human CD44 monoclonal antibody and Cy3-labelled rabbit anti-human CD105+ antibody (eBioscience, San Diego, CA, USA) were added to 100 μl of cell suspension and incubated in the dark at 4 ℃ for 30 minutes. Pre-cooled phosphate buffered saline (PBS) was used to readjust the volume of the cell suspension to 500 μl. A flow cytometer (BD FACSAria, BD Biosciences San Jose, CA, USA) was used to select CD44+/CD105+ HuRCSCs. All cells were resuspended in complete cancer stem cell culture medium: Dulbecco's modified Eagle's medium (DMEM:F12 (HyClone, Logan, UT, USA), supplemented with 10 ng/mL basic fibroblast growth factor, 10 ng/mL epidermal growth factor, 5 μg/mL insulin, 1% bovine serum albumin (BSA) and 5% knockout serum replacement (KnockOut SR) (all from Gibco, Grand Island , NY, USA). The study protocol was approved by the Regional Ethics Committee of Shanghai Geriatric Institute of Chinese Medicine, Shanghai University of Traditional Chinese Medicine (Permission No.: SHAGE-E-202114), in accordance with the 2008 Helsinki declaration.

2.2 3-(4,5-dimethylthiazol-2-yl)-2,5-diphenyltetrazolium-bromide (MTT) assay

Briefly, 2000 cells/ml of each group were seeded in a 96-well plate. After 24 h, 10 μl of MTT solution (Sigma-Aldrich, St. Louis, MO, USA) was added to each group of cells and incubated at 37 ℃ for 3 h. The medium was discarded, 150 μl of dimethyl sulfoxide (DMSO) (Sigma-Aldrich) was added to each well, and the plate was shaken for 15 s to mix well. The culture plate was placed in a microplate reader to record the absorbance value at 450 nm. The formula for calculating the cell proliferation inhibition rate (%) is (1-OD value of experimental group of cells – blank/OD value of control group of cells - blank) × 100%.

2.3 Annexin V-FITC/PI staining and flow cytometric analysis

Briefly, the experiment was performed according to the instruction manual of the Annexin V-FITC Apoptosis Detection Kit (Beyotime Biotechnology, Jiangsu, China). Adherent cells were digested using trypsin, washed with PBS once, centrifuged to remove residual liquid, and gently resuspended in 195 μl of Annexin V-FITC binding solution. Next, 5 μl of Annexin V-FITC was added, and the sample was gently mixed. Finally, 10 μl of propidium iodide (PI) staining solution was added, and the sample was gently mixed and incubated at 20 ℃ in the dark for 30 min. The cells were then detected using a flow cytometer (Cytomics FC 500, Beckman Coulter, Indianapolis, IN, USA).

2.4 Transwell migration assay

Briefly, 200 μl of serum-free medium containing 2000 cells/ml was seeded in the upper chamber of the Transwell insert at 8.0 μm / well. A total of 600 μl of complete medium containing 10 % FBS was inoculated in the chamber below the Transwell insert. The cells were cultured at 37 ℃, 5 % CO_2 for 48 h. Cells that adhered to the membrane surface were fixed using 4 % paraformaldehyde at room temperature for 30 minutes, stained with Sigma-Aldrich Chemical for 5 minutes, rinsed with distilled water twice, and the total number of cells was calculated from three non-overlapping fields under the microscope.

2.5 Capillary tubule formation assay

Briefly, each group of human umbilical vein epithelial cells (HUVECs) were plated on a Matrigel-coated 96-well cell culture plate (2×10^3 cells/well) in the presence and absence of various test substances described in the previous section for the cell migration assay. After 12 h of incubation in a CO_2 incubator, the cells were photographed. To quantitate the data, the number of branch points in three non-overlapping fields was determined.

2.6 RNA extraction and Quantitative real-time reverse transcription PCR(qRT-PCR)

According to the instructions of the RNAprep pure Tissue Kit (TIANGEN Biotech (Beijing) Co., Ltd., Beijing, China), about 20 mg of human tissue samples were taken, added with 800 μl lysis buffer, ground, and homogenized. The

supernatant was retained, added with 200 μl of chloroform, mixed by inversion, and centrifuged at 4 ℃, 13 400 × *g* for 15 min. Two volumes of anhydrous ethanol times were added to the supernatant , mixed by inversion, and centrifuged at 4 ℃, 13400 × *g*, for 30 min. RNA pellet was resuspended with 500 μl 75 % ethanol centrifuged at 4 ℃, 13400 × *g* for 5 min. All the liquid was removed and the RNA pellet was fully dissolved in 300 μl of diethyl pyrocarbonate (DEPC) water. The ratio of OD260/OD280 (generally controlled between 1.8 and 2.0) was detected for 1 μl of the RNA solution to determine the purity and total concentration of RNA. Total RNA was treated with DNase I (Sigma-Aldrich), quantified, and reverse transcribed into cDNA using the ReverTra Ace-α First Strand cDNA Synthesis Kit (TOYOBO). The qRT-PCR was performed with a RealPlex4 real-time PCR detection system from Eppendorf Co. Ltd. (Germany). SYBR Green Real-Time PCR Master Mix (TOYOBO) was used as the fluorescent dye in the nucleic acid amplification. qRT-PCR was completed with 40 amplification cycles as follows: denaturation at 95 ℃ for 15s, annealing at 58 ℃ for 30s, and extension at 72 ℃ for 42s. The relative gene expression levelswere calculated using the $2^{-\Delta\Delta Ct}$ method (ΔCt=Ct_genes–Ct_18sRNA; ΔΔCt=ΔCt_all_groups–ΔCt_blankcontrol_group). The mRNA expression levels were normalised to the expression level of 18s rRNA.

2.7 Western blotting

In brief, the total proteins of each group were subjected to 12% denaturing sodium dodecylsulfate polyacrylamide gel electrophoresis (SDS-PAGE) , and

transferred to a polyvinylidene fluoride (PVDF) membrane (Millipore, Bedford, MA, USA) after completion. After blocking and washing, primary antibodies were added and incubated at 37 ℃ for 45 min. After sufficient washing, the secondary antibodies were added incubated at 37 ℃ for 45 min. The membrane was washed four times with Tris-buffered saline-Tween20 (TBST) at room temperature for 14 min each time. Then, Sigma-Aldrich Chemical was added and the immunoreactive protein bands were developed using an Enhanced Chemiluminescence (ECL) kit (Pierce Biotechnology, Rockford, IL, USA).

2.8 Dot blotting

The total RNAs from each group were spotted on a Hybond-N + membrane. The spotted RNAs were then cross-linked to the membrane using a UV Crosslinker. The membrane was blocked in 5% BSA, and subsequently incubated with rabbit anti-5-hydroxymethylcytidine (hm5C) antibody (Abcam, Cambridge, MA, USA) and horseradish peroxidase (HRP)-conjugated anti-rabbit secondary antibody (Cell Signaling Technology, Danvers, MA, USA), and finally developed using enhanced chemiluminescence reagents and exposed to imaging film [2].

2.9 RNA immunoprecipitation (RIP)-PCR

RIP experiments were performed using the Magna RIP RNA-Binding Protein Immunoprecipitation Kit (Millipore). Briefly, cells from all groups were lysed (500 μl per plate) in a modified cell lysis buffer used for western blotting and IP buffer (20

mM Tris, pH 7.5, 150 mM NaCl, 1% Triton X-100, 1 mM EDTA, sodium pyrophosphate, β-glycerophosphate, Na_3VO_4, and leupeptin; Beyotime institute of Biotechnology). After lysis, each sample was centrifuged to clear the insoluble debris and then pre-incubated with 20 μg of protein A agarose beads (Beyotime institute of Biotechnology) by rocking for 30 min at 4 ℃, followed by centrifugation and transfer to a fresh 1.5 mL tube. Rabbit anti-m6A antibodies (1:100, Abcam) were added and incubated for 90 min before the re-addition of 20 μg of protein A agarose beads to capture the immune complexes. The agarose beads were washed three times using ice-cold homogenization buffer. Then, the co-precipitated RNAs were isolated by resuspending the beads in TRIzol RNA extraction reagent (Thermo Fisher Scientific, Inc., Waltham, MA, USA), and extracted using an RNeasy Mini kit (Qiagen GmbH, Hilden, Germany). Total RNAs were subjected to reverse transcription using a ReverTra Ace-α First Strand cDNA Synthesis kit (Toyobo Life Science, Lausanne Switzerland). PCR amplification was performed for 31 cycles as follows: Denaturation at 95 ℃ for 30 sec, annealing at 65 ℃ for 30 sec and extension at 72 ℃ for 42 sec, using Easy-Load™ PCR Master Mix (Beyotime Institute of Biotechnology). The amplification products were visualized using 1.2% agarose gel electrophoresis [2, 3].

2.10 In vivo xenograft experiments

BALB/$C^{nu/nu}$ mice aged 6–7 weeks and weighing about 20 g were used in the experiment. The BALB/$C^{nu/nu}$ mice were administered with approximately 1×10^5

cells in the log phase. Each experimental group consisted of four mice. After 2 months, the mice were sacrificed, and their tumors were excised. The tumour weight was measured and the tumor volume was calculated according to the formula: Tumor volume (mm^3) = (wh^2)/2, where w is the longest axis (mm) and h is the shortest axis (mm). The animal study was performed at the Shanghai University of Traditional Chinese Medicine with approval from the Institutional Animal Care and Use Committee in accordance with the institutional guidelines. And, all animal experiments complyed with the ARRIVE guidelines and were carried out in accordance with the National Institutes of Health guide for the care and use of Laboratory animals (NIH Publications No. 8023, revised 1978).

2.11 Hematoxylin and eosin (H&E) staining

Tissue samples were fixed using 4 % paraformaldehyde, dehydrated, and embedded in paraffin. Sections were cut to 4 μm and placed on slides. Subsequently, the sections were dewaxed using xylene, followed by ethanol gradient dehydration. Hematoxylin staining solution was added to the sections and incubated at room temperature for 5 min, followed by differentiation using 1 % hydrochloric acid ethanol for 30 sec. Then, ammonia water was added to turn the stained parts blue for 1 min, and the sections were washed using distilled water for 5 min. Subsequently, the eosin staining solution was added to the sections and incubated at room temperature for 2 min, followed by washing with distilled water for 2 min, gradient ethanol decolorization, and xylene penetration for 2 min. Finally, the sections were

coverslipped and sealed with neutral gum.

2.12 Immunofluorescence staining

All fresh tissues were immersed in 4 % paraformaldehyde (Sigma-Aldrich) at room temperature for 30 min. The tissues were then subjected to ethanol gradient dehydration, paraffin embedding, slicing (to a thickness of 6 μm), and xylene dewaxing. The tissue sections were blocked with immunohistochemical blocking solution (Beyotime Biotechnology Co., Ltd.) at 37 °C for 30 min. The blocking solution was discarded and the sections were washed using immunohistochemical cleaning solution (Beyotime Biotechnology Co., Ltd.) three times for 5 min each time at room temperature. Then, primary antibody was added and incubated at 37 °C for 45 minutes. The antibody solution was discarded, and the sections were washed using immunohistochemical cleaning solution (Beyotime Biotechnology Co., Ltd.) three times for 5 min each time at room temperature. Then, the secondary antibody was added and incubated at 37 °C for 45 min. The antibody was discarded, and the sections were washed using immunohistochemical cleaning solution (Beyotime Biotechnology Co., Ltd.) three times for 5 min each time at room temperature. Finally, immunofluorescence blocking solution (Sigma-Aldrich) was added.

2.13 Lipid peroxidation (LPO) assay

The relative malondialdehyde (MDA) concentration in cell or tumor lysates was assessed using a Lipid Peroxidation (MDA) Assay Kit (Abcam, Cambridge, MA,

USA; #ab118970) according to the manufacturer's instructions and previously studies[4, 5]. Briefly, MDA in the sample reacts with thiobarbituric acid (TBA) to generate a MDA-TBA adduct. The MDA-TBA adduct can be quantified colorimetrically (OD = 532 nm). C11-BODIPY dye (Thermo Fisher Scientific, Waltham, MA, USA) was used to detect lipid peroxidation in cells. Oxidation of the polyunsaturated butadienyl portion of the dye results in a shift of the fluorescence emission peak from ~590 to ~510 nm.

2.14 Adenosine triphosphate (ATP) assay

The ATP assay was performed according to the manufacturer's protocol from the Enhanced ATP Assay Kit (Beyotime, Shanghai, China) and according to previous studies [4, 5]. Briefly, 200 μL of the sample lysate was added to 1×106 cells/mL and thoroughly mixed by pipetting. The mixture was then centrifuged at 12,000 × g for 5 minutes at 4 °C, and the supernatant was collected. At the same time, the ATP standard solutions were set up. The ATP standard solutions were adjusted to the following concentrations: 0.01, 0.03, 0.1, 0.3, 1, 3, and 10 μM, respectively, and were tested simultaneously with the samples. Fresh testing solutions were prepared as required by the kit's protocol. The ATP testing solution (100 μL) was added to each of the testing wells and standard wells, and incubated at room temperature for 5 minutes. Then, 20 μL of the test sample or standard solution was added to the wells and quickly mixed. After 5 seconds at room temperature, the relative light unit (RLU) values were measured using a luminometer.

2.15 Iron assay

The measurements of the ferrous iron (Fe2+) were performed using the Iron Analysis Kit (AB83366, Abcam, UK) according to the manufacturer's instructions. Briefly, firstly, cells were immediately homogenized with 5 volumes of Iron Assay buffer. The insoluble material was wiped off via the centrifugation at 13000×g under 4°C, to obtain the supernatant for the assay. For estimation of Fe2+ iron, a 50-μL supernatant was incubated with 50 μL of Iron Reducer Assay buffer in a 96-well microplate for 30 min at room temperature. And then 50 μL of Assay buffer was incubated with 200 μL of reagent mix via pipetting in the dark for 30 min at room temperature. Subsequently, 100 μL Iron Probe was added into the standard and test samples, and the thoroughly mixed sample was incubated for 1 h at room temperature darkly. In the end point, the absorbance was determined with the help of microplate reader[6-8].

2.16 Glutathione (GSH/GSSG) assay

The relative GSH concentration in cells was assessed using a GSH/GSSG Ratio Detection Assay Kit (Abcam, #ab205811) according to the manufacturer's instructions. Briefly[9], whole cell was diluted to 1:80 for GSH analysis, serial dilution of GSH and GSSG stock standards were prepared as standards. A one-step fluorimetric reaction of samples with respective assay buffer and probes was incubated for 30 min. The yellow product (5-thio-2-nitrobenzoic acid) was measured spectrophotometrically

at 412 nm.

2.16 Bioinformatic prediction and analysis

A total of 522 patients with renal cell carcinoma patients(T) and 99 non-renal cell carcinoma patients (N) from the Gene Expression Profiling Interactive Analysis (http://gepia.cancer-pku.cn/index.html), GEPIA, were included in the study patient cohorts. The data for the above patient cohorts were used in gene expression profile analysis, pathological stage plot analysis, multiple gene comparison analysis, and gene correlation analysis using the GEPIA online tool [10].

2.13 Statistical analysis

Each experiment was performed as least three times; data are presented as the mean ± the standard error (SE) where applicable. Differences were evaluated using Student’s t-tests. P values < 0.05 were considered statistically significant. With respect tothe ANOVA and limma options, genes with a |log2FC| cutoff > 1 and q < 0.01 relative to pre-set thresholds were considered to be differently expressed genes (DEGs).

References

1. Si Y, Liu J, Shen H, Zhang C, Wu Y, Huang Y, et al. Fisetin decreases TET1 activity and CCNY/CDK16 promoter 5hmC levels to inhibit the proliferation and invasion of renal cancer stem cell. Journal of cellular and molecular medicine. 2019; 23: 1095-105.
2. Fei X, Cai Y, Lin F, Huang Y, Liu T, Liu Y. Amniotic fluid mesenchymal stem cells repair mouse corneal cold injury by promoting mRNA N4-acetylcytidine modification and ETV4/JUN/CCND2 signal axis activation. Human cell. 2021; 34: 86-98.

3. Ye W, Ni Z, Yicheng S, Pan H, Huang Y, Xiong Y, et al. Anisomycin inhibits angiogenesis in ovarian cancer by attenuating the molecular sponge effect of the lncRNAMeg3/miR421/PDGFRA axis. International journal of oncology. 2019; 55: 1296-312.
4. Huang Y, Lin J, Xiong Y, Chen J, Du X, Liu Q, et al. Superparamagnetic Iron Oxide Nanoparticles Induce Ferroptosis of Human Ovarian Cancer Stem Cells by Weakening Cellular Autophagy. Journal of biomedical nanotechnology. 2020; 16: 1612-22.
5. Wu W, Geng Z, Bai H, Liu T, Zhang B. Ammonium Ferric Citrate induced Ferroptosis in Non-Small-Cell Lung Carcinoma through the inhibition of GPX4-GSS/GSR-GGT axis activity. International journal of medical sciences. 2021; 18: 1899-909.
6. Zhang HY, Zhang BW, Zhang ZB, Deng QJ. Circular RNA TTBK2 regulates cell proliferation, invasion and ferroptosis via miR-761/ITGB8 axis in glioma. European review for medical and pharmacological sciences. 2020; 24: 2585-600.
7. Han F, Li S, Yang Y, Bai Z. Interleukin-6 promotes ferroptosis in bronchial epithelial cells by inducing reactive oxygen species-dependent lipid peroxidation and disrupting iron homeostasis. Bioengineered. 2021; 12: 5279-88.
8. Tang B, Zhu J, Li J, Fan K, Gao Y, Cheng S, et al. The ferroptosis and iron-metabolism signature robustly predicts clinical diagnosis, prognosis and immune microenvironment for hepatocellular carcinoma. Cell communication and signaling : CCS. 2020; 18: 174.
9. Wang K, Zhang Z, Tsai HI, Liu Y, Gao J, Wang M, et al. Branched-chain amino acid aminotransferase 2 regulates ferroptotic cell death in cancer cells. Cell death and differentiation. 2021; 28: 1222-36.
10. Tang Z, Li C, Kang B, Gao G, Li C, Zhang Z. GEPIA: a web server for cancer and normal gene expression profiling and interactive analyses. Nucleic acids research. 2017; 45: W98-W102.
